# Association between maternal and fetal inflammatory biomarkers and offspring weight and BMI during the first year of life in pregnancies with GDM: MySweetheart study

**DOI:** 10.3389/fendo.2024.1333755

**Published:** 2024-05-10

**Authors:** Maria-Christina Antoniou, Dan Yedu Quansah, Leah Gilbert, Amar Arhab, Sybille Schenk, Alain Lacroix, Bobby Stuijfzand, Antje Horsch, Jardena Jacqueline Puder

**Affiliations:** ^1^ Unit of Pediatric Endocrinology and Diabetology, Pediatric Service, Woman-Mother-Child Department, Lausanne University Hospital, Lausanne, Switzerland; ^2^ Obstetric Service, Woman-Mother-Child Department, Lausanne University Hospital, Lausanne, Switzerland; ^3^ Nepean Clinical School, Faculty of Medicine and Health, The University of Sydney, Penrith, NSW, Australia; ^4^ Institute of Higher Education and Research in Healthcare (IUFRS), University of Lausanne, Lausanne, Switzerland; ^5^ Neonatology Service, Woman-Mother-Child Department, Lausanne University Hospital, Lausanne, Switzerland

**Keywords:** gestational diabetes, offspring anthropometry, perinatal inflammation, cord blood CRP, maternal pro-inflammatory cytokines

## Abstract

**Background:**

Gestational Diabetes Mellitus (GDM) is frequently associated with chronic, low-grade inflammation. Whether this environment affects offspring anthropometry during early childhood remains to be elucidated. The aim of this study was to investigate the associations between maternal and fetal (cord blood-umbilical artery) inflammatory biomarkers and offspring weight and BMI up to 1 year in pregnancies with GDM.

**Methods:**

In this prospective secondary analysis of the MySweetheart study, we included 193 women with GDM and their offspring. Maternal and fetal (N=39) predictors included serum levels of inflammatory biomarkers including CRP, IL-6, and TNF-α at 24-32 weeks of gestational age (GA) and in the cord blood. Offspring outcomes were small and large for gestational age (SGA, LGA), sex- and age-adjusted weight, and BMI at birth and at 1 year. Univariate and multivariate regression models were performed. Associations were adjusted for maternal pre-pregnancy BMI, age, and ethnicity.

**Results:**

Mean maternal age was 33.6 ± 4.8 years, and pre-pregnancy BMI 25.9 ± 5.6 kg/m^2^. Their mean gestational age at the 1^st^ GDM visit was 29 ± 2.4 weeks. Gestational age at delivery was 39.7 ± 1.1 weeks, with a mean birthweight of 3.4 ± 0.46 kg; 11.8% of offspring were LGA and 10.8% were SGA. At 1 year of age, mean offspring weight was 9.8 ± 1.2 kg and BMI z-score 0.23 ± 1.1 kg/m^2^. In the models including only maternal predictors, TNF-α at 24-32 weeks of GA was positively associated with SGA and inversely with offspring weight and BMI at birth and at 1 year (p ≤0.034). In the models including only fetal predictors and the combined model, CRP was inversely associated with BMI at 1 year (p ≤0.020).

**Conclusions:**

In women with GDM, maternal and fetal inflammatory biomarkers distinctively influenced offspring anthropometry during the first year of life, independent of maternal age, prepregnancy BMI and ethnicity. These results suggest that low-grade inflammation during pregnancy may affect the developing offspring by leading to a decrease in weight and BMI and may have implications for future personalized follow-up of women with GDM and their offspring.

## 1 Introduction

Inflammation is associated with an increased risk of insulin resistance, hyperglycemia, metabolic syndrome, and cardiovascular disease (1–3). Increased inflammation during pregnancy caries an increased risk for short-term complications in the mother and the offspring, including miscarriage, gestational diabetes mellitus (GDM), preeclampsia, preterm birth, intrauterine growth restriction, and birth defects (4–8). Long-term complications in the offspring such as specific behavioral complications, and psychiatric disorders have also been recently evoked (9). Maternal GDM may be associated with a state of chronic, low-grade inflammation, which often precedes its diagnosis (10). The complex relationship between gestational diabetes and inflammation is underscored by evidence of increased plasma levels of pro-inflammatory cytokines, including plasma C-reactive protein (CRP), interleukin-6 (IL-6), tumor necrosis factor alpha (TNF-α) and/or IL-1a, in women with GDM and their fetuses (umbilical cord) in some, but not all studies (11–16).

To the best of our knowledge, the association between maternal inflammatory biomarkers during pregnancy and offspring weight or BMI during the 1^st^ year of life in pregnancies with GDM has not been previously investigated. Most of the studies in the general population as well as a study including a large population with a wide spectrum of glucose tolerance (Hyperglycemia and Adverse Pregnancy Outcome (HAPO) study), found an inverse association between pro-inflammatory biomarkers during pregnancy and weight and adiposity at birth (17–21). In the general pregnant population, this association was not significant (22). Beyond birth, 2^nd^ trimester plasma CRP in healthy pregnancies has been associated with a higher childhood fat mass index (FMI) and trunk FMI in preschoolers (23). Regarding fetal predictors, existing studies in different populations (the general pregnant population, and populations with high prevalence of GDM), found no association between cord blood inflammatory biomarkers, such as CRP, IL-6 and TNF-α, and offspring weight and adiposity at birth (18, 24–26). However, the association between both maternal or fetal (cord blood) inflammatory biomarkers, including CRP, IL-6, and TNF-α has not been studied in populations with GDM (18, 21). There is also a lack of data on the impact of inflammatory biomarkers during the first year of life beyond birth in this population. Obesity and aging are associated with low-grade inflammation (27–29). Inflammatory biomarkers vary across different ethnic groups,underscoring the intricate interplay of genetic, environmental and social factors in shaping health disparities (30) A recent study in the general population found that maternal obesity-related inflammation during pregnancy increased the risk of childhood obesity in an ethnic-specific manner (31). However, it is unclear if this low-grade inflammation environment in pregnancies with GDM influences offspring anthropometry and contributes to the development of metabolic health disorders in offspring during early childhood as well as later in life.

The aims of this study were: 1) to investigate the associations between maternal and fetal inflammatory biomarkers on offspring anthropometric parameters at birth and at 1 year in a population of women with GDM; 2) to determine if the proposed associations are independent of maternal pre-pregnancy BMI, age, and ethnicity.

## 2 Materials and methods

### 2.1 Study design and informed consent

The present study is a secondary data analysis of the *MySweetheart trial*, a randomized-controlled intervention trial of 211 women ≥18 years with GDM and their offspring (Clinicaltrials.gov NCT02890693) (32), followed during pregnancy until one year postpartum in the Diabetes and Pregnancy Unit in the Lausanne University Hospital, Switzerland. GDM was diagnosed between 24 and 32 weeks of gestational age (GA), according to the International Association of Diabetes and Pregnancy Study Group (IADPSG Criteria) (33). Details of the study protocol have been previously described (32). Women on strict bed rest, with a severe mental health disorder, pre-existing diabetes, and women who did not understand English or French were excluded from the study. Signed informed consent was obtained from all participating women. The study was conducted in accordance with the guidelines of the declaration of Helsinki and good clinical practice. The Human Research Ethics Committee of the Canton de Vaud approved the study protocol (study number 2016-00745). Included women were randomized to the usual care or intervention group after the baseline visit and signing of an informed consent.

### 2.2 Allocation groups

#### 2.2.1 Usual care group

Women randomized to usual care received treatment based on the American Diabetes Association and on the Endocrine Society guidelines for the management of GDM (34, 35). They had regular appointments every 1-3 weeks with a physician or a diabetes-specialist nurse and a dietician after the GDM diagnosis, and were encouraged to increase physical activity (34). During the 1^st^ visit at 24-32 weeks of GA, they were taught how to perform self-monitoring of blood glucose (4 times during the day-fasting and 2 hours post-prandial). If glucose values remained above targets two or more times during a 1 to 2-week period (fasting glucose >5.3 mmol/l, 1-h postprandial glucose >8 mmol/l and/or 2-h postprandial glucose >7 mmol/l) despite lifestyle changes, insulin treatment or very rarely metformin was introduced depending on patient’s glucose values and preferences. After delivery, glucose controls and glucose-lowering treatments were stopped. Women saw a physician and a dietician at the 6-8 week postpartum visit after an oGTT test to discuss further management.

#### 2.2.2 Intervention group

Women randomized to the intervention group received a multidimensional, interdisciplinary lifestyle and psychosocial intervention on top of usual care, centered on eating behavior, a balanced food intake, as well as physical activity and breastfeeding. The intervention also included a psychosocial component, including the assessment and treatment of depression during and after pregnancy. During pregnancy and up to 1 year postpartum, patients were supported by a lifestyle coach [see (32] for more details).

### 2.3 Follow-up

For the current analysis, we used maternal data from their 1^st^ GDM visit at 24-32 weeks of GA, and offspring data at birth, and at 1 year. At the 1^st^ GDM visit, information on maternal socio-demographic characteristics were collected and maternal anthropometric parameters and inflammatory biomarkers were measured in the serum. Immediately after birth, blood was drawn from the umbilical cord to measure inflammatory biomarkers. Newborn anthropometric parameters were obtained from the hospital birth record. At 1 year postpartum, the offspring’s anthropometric measures including weight and length were collected.

### 2.4 Maternal, fetal and offspring parameters

#### 2.4.1 Maternal descriptive and confounder variables

Maternal socio-demographic parameters, including age, ethnicity, and parity were collected during the 1^st^ GDM visit. Ethnicity was classified into Low (Europe, North America) and High Metabolic Risk (Asia, Central and South America, Africa, Oceania) ethnic groups (35). Pre-pregnancy BMI was calculated using weight information from medical charts and height measured during the 1^st^ visit at the GDM clinic. In rare circumstances when pre-pregnancy weight was not mentioned in the chart, it was self-reported during the 1^st^ GDM visit. Height was measured at the 1^st^ GDM visit to the nearest 0.1 cm with a regularly calibrated Seca^®^ height scale. GWG was determined as the difference between the weight at the end of pregnancy and pre-pregnancy weight. Glucose-lowering maternal medical treatment for GDM was classified into two categories (no treatment, treatment with insulin and/or very rarely metformin). HbA1c using a chemical photometric method (conjugation with boronate; Afinion^®^).

#### 2.4.2 Maternal and fetal (cord blood) inflammatory predictor variables

At the 24-32 weeks of GA visit, maternal inflammatory parameters, including CRP, IL-6, and TNF-α were measured in maternal serum. At birth, CRP, IL-6, and TNF-α were again measured in the cord blood (umbilical artery) (36). CRP was analyzed at the Lausanne University Hospital in serum aliquots using a latex-enhanced immunoturbidimetric assay on a Cobas 8000 autoanalyzer (Roche Diagnostics, Mannheim, Germany) with assay characteristics as reported by the manufacturer. We also measured IL-6 (U-PLEX Human IL-6 Antibody Set) and TNF-α (U-PLEX Human TNF-α Antibody Set) using ELISA according to the manufacturer’s instructions.

#### 2.4.3 Offspring descriptive and outcome variables

At birth, weight (g) and length (cm) were documented; percentiles and z-scores for these parameters were calculated using the Intergrowth 21^st^ newborn size application tool (37). BMI was also calculated. LGA was defined as birth weight >90^th^ percentile and SGA as birth weight <10^th^ percentile for sex and gestational age. Gestational age was calculated according to the date of the last menstruation, or as assessed by the fetal ultrasound in the cases where gestational age was adapted during the early in-utero ultrasound evaluation. Neonatal antropometric parameters were obtained from patient medical charts. If the birth took place in another hospital or clinic, they were provided by the respective hospital.

At the 1 year visit, offspring weight (kg) and length (cm) were measured, using standardized methods (38). BMI was calculated. Z-scores for weight, length and BMI were calculated using the WHO Anthro Survey Analyser tool -Offline version (39).

#### 2.4.4 Predictors and outcomes

Predictors comprised maternal (1^st^ GDM visit) and fetal (cord blood), including CRP, IL-6, and TNF-α. Outcomes included offspring anthropometric parameters at birth and 1 year. More precisely, birth outcomes included weight, BMI, LGA, and SGA, and outcomes at 1 year, weight, and BMI.

### 2.5 Statistical analysis

Data analysis was performed using Stata/SE 16.0 (StataCorp LLC, TX, USA). The normality of continuous variables were assessed using histograms and Q-Q plots. Outcomes variables were normally distributed. Continuous variables were described as means and standard deviations and binary outcomes as N (percentages) (**Table 1**). Comparisons between the intervention and control group were conducted using the unpaired t-test for normally distributed continuous variables, the Mann-Whitney test for continuous variables with non-normal distribution and the Fisher’s exact test for binary variables. In all analyses, predictors and outcomes did not differ in the respective allocation groups (intervention vs usual care) and the effect sizes were similar. Thus, women from both groups were pooled together and we adjusted for group allocation in all analyses. Where appropriate, all analyses were also adjusted for infant age and sex.

**Table 1:** Maternal and offspring characteristics.

Maternal Characteristics		Infant characteristics	
Number of patients (N)	193	**Birth anthropometric parameters**	
Age (years)	33.6 ± 4.8	Number of patients	190 (Male:52%)
High risk ethnicity (yes; N(%)	39 (22.7%)	Gestational age (weeks)	39.7 ± 1.1
Pre-pregnancy BMI (kg/m^2^)	25.9 ± 5.6	Weight (kg)	3.4 ± 0.46
Gestational weight gain (kg)	12.6 ± 6.5	Weight z-score (SD) ^1^	0.18 ± 1.1
Glucose lowering medical treatment	85 (46.5%)	Length (cm)	49.6 ± 2.4
Gestational age at the at the 1^st^ GDM visit (weeks)	29 ± 2.4	Length z-score (SD) ^1^	0.10 ± 1.4
HbA1c at the 1^st^ GDM visit (%)	5.1 ± 0.31	BMI (kg/m^2^)	13.7 ± 1.7
(mmol/mol)	32.2 ± 2.0	LGA ^1,2^	22 (11.8%)
**Maternal inflammatory parameters**		SGA ^1,3^	20 (10.8%)
CRP at the 1^st^ GDM visit (mg/L)	4.5 ± 3.8	**1 year anthropometric parameters**	
IL-6 at the 1^st^ GDM visit (pg/ml)	1.0 ± 1.3	Number of patients	170 (Male:52%)
TNF-α at the 1^st^ GDM visit (pg/ml)	0.74 ± 0.76	Age (months)	12.4 ± 1.0
Fetal parameters		Weight (kg)	9.8 ± 1.2
Number of patients	39	Weight z-score (SD) ^4^	0.32 ± 0.91
Cord blood CRP (mg/L)	0.29 ± 0.51	Length z-score (SD) ^4^	0.27 ± 1.2
Cord blood IL-6 (pg/ml)	7.21 ± 7.55	BMI (kg/m^2^)	16.9 ± 1.6
Cord blood TNF-α (pg/ml)	1.5 ± 0.46	BMI z-score (SD) ^4^	0.23 ± 1.1

BMI, body mass index; CRP, C-reactive protein; GDM, gestational diabetes mellitus; HbA1c, glycated hemoglobin; IL-6, interleukin 6; LGA, large for gestational age; SD, standard deviation; SGA, small for gestational age; TNF-α, tumor necrosis factor alpha.

^1^according to the Intergrowth 21^st^ newborn size application tool (37).

^2^LGA: birth weight >90th percentile for sex and gestational age using the Intergrowth 21^st^ newborn size application tool (37).

^3^SGA: birth weight <10th percentile for sex and gestational age using the Intergrowth newborn size application tool (37).

^4^according to the WHO Anthro Survey Analyser tool (39).

We performed a Spearman’s rank correlation coefficient test to investigate the correlation between maternal and fetal cord inflammatory parameters and the presence of collinearity (**Supplementary Table 1**). No collinearity was found between predictors (r_s_ < 0.6). We then conducted univariate linear and logistic regression analyses using offspring outcomes as the dependent variables (**Supplementary Tables 2**, **3**). Maternal and fetal predictors with a p-value < 0.05 in univariate analysis were included in stepwise multiple regression analyses models. We performed different multivariate models. In terms of predictors, three multivariate models were used, a first model including only maternal predictors, a second model including only fetal predictors and a third model including both maternal and fetal predictors. Fetal predictors were available for N = 39 participants. In terms of adjustments, the above models were adjusted for maternal age, pre-pregnancy BMI, and ethnicity in addition to the already mentioned adjustments (group allocation, offspring age and sex). The analyses and adjustments were performed in order to identify the most significant maternal and fetal predictors of infant anthropometric parameters at birth and 1 year, and to determine the extent of the impact of these predictors independent of maternal confounder variables, i.e., ethnicity, age, and obesity; known to be associated with low-grade inflammation (27–29) (**Table 2**). For all analyses, β-coefficients (for continuous outcomes) and adjusted odds ratios (aORs-for binary outcomes) are reported along with their 95% confidence intervals (CIs), and statistical significance was set at p<0.05.

**Table 2:** Maternal serum and fetal cord blood predictors of offspring anthropometric outcomes in multivariate regression analyses.

Offspring outcomes	Predictors	OR^1^/β-coefficient	95% CI		p-value
Birth					
	**Maternal**				
Weight (kg)	TNF-α at the 1^st^ GDM visit (pg/ml) ^2^	-0.090	-0.170	-0.010	0.028
BMI (kg/m2)	TNF-α at the 1^st^ GDM visit (pg/ml) ^2^	-0.455	-0.773	-0.137	0.005
SGA ^3^	TNF-α at the 1^st^ GDM visit (pg/ml) ^2^	1.609 ^1^	1.036	2.500	0.034
1 year					
	**Maternal & Fetal**				
BMI (kg/m2)	Cord blood CRP (mg/L)	-2.838	-4.029	-1.646	0.001
	**Maternal**				
Weight (kg)	TNF-α at the 1^st^ GDM visit (pg/ml) ^2^	-0.411	-0.659	-0.163	0.001
BMI (kg/m2)	TNF-α at the 1^st^ GDM visit (pg/ml) ^2^	-0.702	-1.069	-0.335	0.000
	**Fetal**				
BMI (kg/m2)	Cord blood CRP (mg/L)	-2.838	-4.029	-1.646	0.001

BMI, body mass index; CI, Confidence Interval; CRP, C-reactive protein; GDM, gestational diabetes mellitus; HbA1c, glycated hemoglobin; IL-6, interleukin 6; LGA, large for gestational age; OR, Odds Ratio; SD, standard deviation; SGA, small for gestational age; TNF-α, tumor necrosis factor alpha.

^1^this value corresponds to an OR.

^2^the mean gestational age at the 1^st^ GDM visit was 29 ± 2.4 weeks.

^3^SGA: birth weight < 10^th^ percentile for sex and gestational age using the Intergrowth 21st newborn size application tool (37).

Stepwise multiple logistic regression analyses. All analyses were adjusted for maternal age, pre-pregnancy BMI, and ethnicity (high/low risk), allocation group (intervention/usual care), and infant sex and age (where appropriate). Outcomes are only shown if at least one predictor is found. Only significant results are displayed (defined significance, p-value <0.05, see text). Three distinct sub-models were performed (combined model, including maternal and fetal predictors, model including only maternal or only fetal predictors), and results are displayed separately.

## 3 Results

The initial population included 211 women with GDM. As previously described (38), one woman was excluded as the diagnosis of GDM was done too early (< 13 weeks of gestation), and 17 were excluded due to multiple gestation, and/or because their offspring were premature (gestational age (GA) < 37 weeks. Thus, 193 women and their offspring were included in the analyses.

### 3.1 Maternal, fetal and infant characteristics

**Table 1** describes the maternal characteristics, fetal inflammatory parameters, and offspring anthropometry at birth and 1 year. In summary, mean maternal age was 33.6 ± 4.8 years, and pre-pregnancy BMI was 25.9 ± 5.6 kg/m^2^. Their mean gestational age at the 1^st^ GDM visit at 24 to 32 weeks of GA was 29 ± 2.4 weeks. Gestational age at delivery was 39.7 ± 1.1 weeks, with a mean birthweight of 3.4 ± 0.46 kg; 11.8% of offspring were LGA and 10.8% were SGA. At 1 year of age, mean offspring weight was 9.8 ± 1.2 kg and BMI z-score 0.23 ± 1.1 kg/m^2^. Maternal and fetal (cord blood) inflammatory biomarkers, including CRP, IL-6, and TNF-α were not significantly correlated (**Supplementary Table 1**).

### 3.2 Associations between maternal and fetal predictors and offspring anthropometry at birth and 1 year in univariate analyses

#### 3.2.1 Birth

TNF-α at the 1^st^ GDM visit was inversely associated with offspring weight [β-coefficient= -0.107 (CI: -0.189; -0.026), p=0.010] and BMI at birth [β-coefficient= -0.501 (CI: -0.816; -0.186), p=0.002] and positively with SGA [OR= 0.492 (CI: 0.056; 0.927), p=0.027], and cord blood CRP was inversely associated with offspring weight [β-coefficient= -0.370 (CI: -0.659; -0.081), p=0.015] and BMI at birth [β-coefficient= -1.052 (CI: -1.900; -0.205), p=0.017]. No association was found between maternal and fetal inflammatory biomarkers and LGA (all p ≥0.199, **Supplementary Table 2**).

#### 3.2.2 One year

TNF-α at the 1^st^ GDM visit was inversely associated with offspring weight [β-coefficient= -0.376 (CI: -0.625; -0.128), p=0.003] and BMI at 1 year pβ-coefficient= -0.641 (CI: -1.003; -0.279), p=0.001], and cord blood CRP and TNF-α were inversely associated with offspring BMI at 1 year [β-coefficient= -2.566 (CI: -3.666; -1.465), p=0.000] and [β-coefficient= -2.177 (CI: -3.671; -0.684), p=0.006], respectively, **Supplementary Table 3**).

### 3.3 Associations between maternal and fetal predictors and offspring anthropometry at birth and 1 year in multivariate analyses

The significant results of all multivariate analyses are shown in **Table 2**.

#### 3.3.1 Birth

In the models including only maternal predictors, TNF-α at the 1^st^ GDM visit was inversely associated with offspring weight [β-coefficient= -0.090 (CI: -0.170; -0.010), p=0.028] and BMI [β-coefficient= -0.455 (CI: -0.773; -0.137), p=0.005], and positively with SGA [OR= 1.609 (CI: 1.036; 2.500), p=0.034]. No significant associations were found between maternal and fetal inflammatory biomarkers and offspring anthropometry in models including only fetal predictors or in the combined model (p ≥ 0.05).

#### 3.3.2 One year

In models including maternal predictors, TNF-α at the 1^st^ GDM visit was inversely associated with offspring weight [β-coefficient= -0.411 (CI: -0.659; -0.163), p=0.001] and BMI at 1 year [β-coefficient= -0.702 (CI: -1.069; -0.335), p=0.000]. In models including fetal predictors as well as in the combined model, cord blood CRP showed an inverse association with BMI at 1 year [β-coefficient= -2.838 (CI: -4.029; -1.646), p=0.001].

## 4 Discussion

This prospective, secondary analysis of the MySweethheart study, of women with GDM and their offspring showed that maternal and fetal inflammatory biomarkers predicted offspring weight and BMI during the 1^st^ year of life, independent of maternal age, pre-pregnancy BMI, and ethnicity. In the adjusted analyses, maternal TNF-α at 24-32 weeks GA was positively associated with SGA and inversely with offspring weight and BMI at birth and at 1 year, whereas. CRP was inversely associated with offspring BMI at 1 year independent of maternal predictors. Thus, while maternal cytokines predicted lower weight and BMI, both at birth and up to 1 year, the impact of fetal CRP was observed at 1 year.

### 4.1 Impact of maternal and fetal inflammatory parameters on offspring anthropometry at birth

In our study, maternal serum concentrations of TNF-α at the 1^st^ GDM visit at 24-32 weeks of GA were negatively associated with offspring weight and BMI at birth and positively with SGA. This is consistent with a recent study in a healthy population that found a negative correlation between inflammatory biomarkers such as CRP, and IL-6, and birthweight (17). Other studies have evaluated the impact of maternal inflammatory parameters and offspring birth outcomes in healthy pregnant women, as well as in populations with some degree of glucose intolerance (19, 20, 22, 40, 41). Thus, maternal CRP levels in early pregnancy were associated with higher rates of SGA in the general population (40), and those during the 3^rd^ trimester were inversely associated with offspring weight and sum of skinfolds at birth in the general population and in a subpopulation of the HAPO Study (19, 20). No correlations between maternal 3^rd^ trimester TNF-α, and IL-6 values and fetal adiposity or birthweight were found in a healthy pregnant population of women without GDM (22), whereas maternal 2^nd^ and 3^rd^ trimester IL-6 values were inversely associated with weight and sum of skinfolds at birth in a study including women with GDM and without GDM (41). A study performed in women with a previous history of having a macrosomic infant, but without GDM, found no association between maternal IL-6 and TNF-α and birthweight and adiposity measures in the total or male cohort; however, an inverse association was found between maternal 3^rd^ trimester IL-6 levels and the sum of skinfolds at birth in the female cohort (18). Thus, most of the previous studies have shown an inverse association between maternal pro-inflammatory markers, including CRP, IL-6 and/or TNF-α, and offspring anthropometry at birth. For the first time, our data extends this now to a metabolically high-risk population of women with GDM. Evidence indicates a U-shaped curve relating the size at birth with long-term cardiometabolic disease, highlighting the significance of optimal fetal growth for long-term health outcomes (42, 43). The impact of maternal pro-inflammatory factors on fetal growth may be mediated by their direct and indirect effects on the placental development, function and immunomodulatory activity, as well as on the hormone synthesis and action in the materno-fetal unit (41, 44, 45). The absence of association between IL-6 levels and BMI or weight in our study may be possibly explained by the fact that IL-6 correlates more closely with visceral adiposity (46), which was not specifically evaluated.

We found no associations between cord blood inflammatory biomarkers and offspring anthropometry at birth. Similarly, studies in the general population as well as in populations with high prevalence of GDM, and/or pre-existing diabetes, found no association between cord blood IL-6, CRP or increased CRP [CRP >0.3 mg/l (2.9 nmol/l)] levels and one or more anthropometric/adiposity measures at birth (including weight, weight z-score, LGA, fat mass, % body fat, sum of skinfold thickness) (21, 24–26).During the process of birth, there is a physiological increase in the cord blood pro-inflammatory marker concentration and this increase may be further influenced by other parameters, including the duration of labor and the mode of delivery (47, 48).Therefore, cord blood inflammatory biomarkers may be a complex reflexion of both the prenatal and the perinatal fetal inflammatory milieu, explaining the correlation between inflammatory biomarkers in the cord blood and offspring anthropometry later in life (at 1 year) but not at birth. Yeung et al, found an association between cord blood CRP levels and DNA methylation in the cord blood, particularly in gene regions associated with angiogenic and inflammatory pathways, which in turn could have an impact on future cardio-metabolic risk (49). Recent research suggests that methylation in specific inflammation-related genes in the cord blood is associated with adiposity measures later in the development, such as in early childhood (50). Additional investigation is warranted to elucidate the complex mechanisms that underlie the impact of pro-inflammatory cytokines in the cord blood on offspring body composition and metabolic profile during childhood.

### 4.2 Impact of maternal and fetal metabolism on infant weight and BMI during childhood

In our population, maternal serum concentrations of TNF-α during the 3^rd^ trimester were inversely associated with offspring weight and BMI at 1 year. Our results are in agreement with a study in a population of women with a relatively low GDM prevalence (4.5%), where higher 2^nd^ trimester CRP levels were associated with a higher childhood fat mass index (FMI), and trunk FMI, but this later in the development, i.e. in early (3-5 years) and mid childhood (7-10 years) (23). Our results are, however, contrary to a study performed in the general population where 1^st^ trimester IL-6 and TNF-α were not associated with offspring adiposity measures, including weight, BMI z-scores at 2-6 years (31). In another study, 1^st^ trimester IL-6 and TNF-α were not associated with weight and adiposity measures at 6 months of age in the total and male cohort, but only with adiposity measures in the female cohort (18). In a large, Danish general population, CRP, TNF-α, IL-6, and IL-1b measures in the 3^rd^ trimester was not associated with offspring BMI, waist circumference, blood pressure, glucose metabolism measures, or lipid profile at the age of 20 years (51).

Data on the association between fetal (cord blood) inflammatory parameters and offspring growth during the first years of life are scarce. The above mentioned study of women with a history of macrosomia in the absence of GDM, found no association between cord blood IL-6, and TNF-α and offspring weight or adiposity measures at 6 months in their cohort (18). Regarding fetal predictors, in a cohort of children born extremely premature (< 28 weeks gestational age), showed that elevated IL-6 on day 1 in the newborn was associated with an increased risk for obesity at 2 years in multivariate models (52).

### 4.3 Speculations on the mechanisms of impact of maternal and fetal metabolism on offspring anthropometry during the first years of life in women with GDM

In pregnancies with GDM, the impact on offspring anthropometry is multifaceted, involving intricate mechanisms that span maternal, placental, and fetal domains (38, 53, 54). These include various factors such as maternal insulin resistance, elevated cytokines, and subsequent effects on placental function (41, 44). Inflammation and hyperglycemia lead to disruptions in insulin-like growth factors and adiponectin influencing fetal growth patterns and adipose tissue development (55, 56). Exposure to GDM may induce epigenetic modifications, affecting gene expression linked to fetal development and could be a mechanism leading to metabolic dysfunction later in life (57). Additionally, maternal obesity and excessive gestational weight gain, prevalent in GDM, independently contribute to adverse offspring outcomes (58, 59). This collective interplay underscores the complexity of factors influencing offspring anthropometry in the context of GDM, emphasizing the need for a comprehensive understanding of both inflammatory and metabolic pathways in order to identify potential therapeutic targets.

### 4.4 Strengths and limitations

To our knowledge, this is the first study assessing the impact of maternal and fetal inflammatory biomarkers on offspring anthropometric parameters at birth and up to 1 year of life in a metabolically high-risk population of women with GDM and their offspring. Another strength of our study is the prospective design. Nevertheless, some limitations ought to be mentioned. Maternal inflammatory biomarkers were measured only once during gestation. Moreover, offspring predictors did not include height and head circumference as we opted to focus on metabolic health-related anthropometric parameters. As the focus of this study was to investigate the relationship between biomarkers and offspring metabolic health-related offspring in a metabolically high-risk population, we lack a proper control group of healthy participants. Cord blood (umbilical artery) parameters were only available for 39 patients; which could influence our results, especially the correlations. Additionally, due to the small sample size of cord blood parameters, separate analyses for the intervention and usual care group, as well as infant sex could not be performed. However, maternal and fetal predictors and infant anthropometry did not differ between both groups and we always adjusted for group allocation.

### 4.5 Conclusions

Maternal and fetal inflammatory biomarkers including TNF-α at 24-32 weeks of GA and cord blood CRP distinctively influenced offspring weight and BMI during the first year of life, and this independent of maternal age, ethnicity, and pre-pregnancy BMI. The impact of fetal inflammatory biomarkers was not apparent at birth but was observed at 1 year. Further research is warranted to elucidate how the inflammatory milieu before and during birth might impact the developing offspring, and may in turn have implications for designing intervention strategies based on maternal and cord blood inflammatory biomarkers in order to decrease the risk of offspring metabolic dysfunction in the medium and long-term.

## MySweetHeart Research Group

AA, Pascal Bovet, Arnaud Chiolero, Stefano Di Bernardo, Adina Mihaela Epure, Sandrine Estoppey Younes, LG, Justine Gross, AH, Stefano Lanzi, Seyda Mayerat, Yvan Mivelaz, JP, DQ, Jean-Benoit Rossel, Nicole Sekarski, Umberto Simeoni, BS, Yvan Vial.

## Data availability statement

The datasets presented in this article are not readily available because many of them are still being worked on but are available from the corresponding author on reasonable request. Requests to access the datasets should be directed to jardena.puder@chuv.ch.

## Ethics statement

The study was approved by the Human Research Ethics Committee of the Canton de Vaud (study number 2016-00745). The study was conducted in accordance with the local legislation and institutional requirements. Written informed consent for participation in this study was obtained from all participating women (for themselves and their offspring).

## Author contributions

M-CA: Formal analysis, Writing – original draft. DYQ: Investigation, Methodology, Writing – review & editing. LG: Investigation, Methodology, Writing – review & editing. AA: Investigation, Methodology, Writing – review & editing. SS: Methodology, Writing – review & editing. AL: Methodology, Writing – review & editing. BS: Methodology, Writing – review & editing. AH: Conceptualization, Funding acquisition, Writing – review & editing. JJP: Conceptualization, Funding acquisition, Project administration, Supervision, Writing – review & editing.
