# Supplementary material for: Association between maternal and fetal inflammatory biomarkers and offspring weight and BMI during the first year of life in pregnancies with GDM: MySweetheart study
Source: Front Endocrinol (Lausanne). 2024 May 10;15:1333755. doi: 10.3389/fendo.2024.1333755 (PMC11116579; doi:10.3389/fendo.2024.1333755)
Supplement: Supplementary file 1 [file Table_1.docx]

Supplementary table 1. Correlations between maternal and fetal inflammatory biomarkers

| **Cord blood inflammatory biomarkers** | | | |
| --- | --- | --- | --- |
|  |  |  |  |
| Variable | CRP (mg/L) | IL-6 (pg/ml) | TNF-α  (pg/ml) |
| **Maternal inflammatory biomarkers at the 1^st^ GDM visit** |  |  |  |
| CRP (mg/L) | 0.1484 | -0.1082 | 0.1995 |
| p-value | 0.4993 | 0.5425 | 0.2434 |
| IL-6 (pg/ml) | 0.0571 | 0.0619 | 0.0613 |
| p-value | 0.7774 | 0.716 | 0.7107 |
| TNF-α (pg/ml) | 0.1715 | 0.2414 | -0.0687 |
| p-value | 0.3923 | 0.156 | 0.6818 |

Abbreviations: *CRP* C-reactive protein; *GDM* gestational diabetes mellitus; *IL-6* interleukin 6; *TNF-a* tumor necrosis factor alpha.

Correlation analyses between maternal and fetal inflammatory biomarkers using the Spearman’s rank correlation coefficient test

Supplementary table 2. Maternal and fetal predictors of anthropometry at birth in univariate regression analysis

| **Birth Anthropometric Parameters** |  | **Predictors** | **OR ^3^/** | **95% CI** | | **p-value** |
| --- | --- | --- | --- | --- | --- | --- |
|  |  |  | **Beta-Coefficient** |  |  |  |
| Weight (kg) | Maternal Predictors | CRP at the 1^st^ GDM visit (mg/L) | 0.011 | -0.007 | 0.029 | 0.223 |
|  |  | IL-6 at the 1^st^ GDM visit (pg/ml) | -0.013 | -0.059 | 0.033 | 0.585 |
|  |  | TNF-α at the 1^st^ GDM visit (pg/ml) | -0.107 | -0.189 | -0.026 | 0.010 |
|  | Fetal Predictors | Cord blood CRP (mg/L) | -0.370 | -0.659 | -0.081 | 0.015 |
|  |  | Cord blood IL-6 (pg/ml) | -0.009 | -0.029 | 0.011 | 0.379 |
|  |  | Cord blood TNF-α (pg/ml) | -0.044 | -0.369 | 0.280 | 0.784 |
| BMI (kg/m^2^) | Maternal Predictors | CRP at the 1^st^ GDM visit (mg/L) | 0.037 | -0.032 | 0.106 | 0.288 |
|  |  | IL-6-α at the 1^st^ GDM visit (pg/ml) | -0.080 | -0.259 | 0.099 | 0.377 |
|  |  | TNF-α at the 1^st^ GDM visit (pg/ml) | -0.501 | -0.816 | -0.186 | 0.002 |
|  | Fetal Predictors | Cord blood CRP (mg/L) | -1.052 | -1.900 | -0.205 | 0.017 |
|  |  | Cord blood IL-6 (pg/ml) | -0.041 | -0.106 | 0.023 | 0.203 |
|  |  | Cord blood TNF-α (mg/ml) | -0.502 | -1.549 | 0.550 | 0.546 |
| LGA ^1^ | Maternal Predictors | CRP at the 1^st^ GDM visit (mg/L) ^3^ | -0.007 | -0.130 | 0.117 | 0.916 |
|  |  | IL-6 at the 1^st^ GDM visit (pg/ml) ^3^ | 0.151 | -0.080 | 0.383 | 0.199 |
|  |  | TNF-α at the 1^st^ GDM visit (pg/ml) ^3^ | -0.500 | -1.490 | 0.488 | 0.322 |
|  | Fetal Predictors | Cord blood CRP (mg/L) ^3^ | -6.448 | -20.131 | 7.235 | 0.356 |
|  |  | Cord blood IL-6 (pg/ml) ^3^ | -0.001 | -0.152 | 0.150 | 0.990 |
|  |  | Cord blood TNF-α (mg/ml) ^3^ | -1.357 | -4.715 | 2.000 | 0.428 |
| SGA ^2^ | Maternal Predictors | CRP at the 1^st^ GDM visit (mg/L) ^3^ | 0.004 | -0.124 | 0.131 | 0.953 |
|  |  | IL-6 at the 1^st^ GDM visit (pg/ml) ^3^ | 0.155 | -0.078 | 0.387 | 0.192 |
|  |  | TNF-α at the 1^st^ GDM visit (pg/ml) ^3^ | 0.492 | 0.056 | 0.927 | 0.027 |
|  | Fetal Predictors | Cord blood CRP (mg/L) ^3^ | 0.270 | -1.212 | 1.753 | 0.721 |
|  |  | Cord blood IL-6 (pg/ml) ^3^ | 0.015 | -0.095 | 0.125 | 0.783 |
|  |  | Cord blood TNF-α (pg/ml) ^3^ | -0.200 | -2.095 | 1.694 | 0.836 |

Abbreviations: *BMI* body mass index; *CI* Confidence Interval; *CRP* C-reactive protein; *GDM* gestational diabetes mellitus; *IL-6* interleukin 6; LGA large for gestational age; *OR* Odds Ratio; SGA small for gestational age; *TNF-a* tumor necrosis factor alpha.

^1^ LGA: birth weight >90th percentile for sex and gestational age using the Intergrowth 21^st^ newborn size application tool (37)

^2^ SGA: birth weight ˂10th percentile for sex and gestational age using the Intergrowth newborn size application tool (37)

^3^ this value corresponds to an OR

Univariate linear and logistic regression analyses at birth, adjusted for group allocation (intervention/usual care) and infant sex and gestational age (where appropriate).

Supplementary table 3. Maternal and fetal predictors of anthropometry at 1 year in univariate regression analysis

| **1 year Anthropometric Parameters** |  | **Predictors** | **Beta-Coefficient** | **95% CI** | | **p-value** |
| --- | --- | --- | --- | --- | --- | --- |
|  |  |  |  |  |  |  |
| Weight (kg) | Maternal Predictors | CRP at the 1st GDM visit (mg/L) | 0.001 | -0.052 | 0.054 | 0.972 |
|  |  | IL-6 at the 1st GDM visit (pg/ml) | -0.065 | -0.181 | 0.051 | 0.268 |
|  |  | TNF-α at the 1st GDM visit (pg/ml) | -0.376 | -0.625 | -0.128 | 0.003 |
|  | Fetal Predictors | Cord blood CRP (mg/L) | 0.424 | -2.685 | 3.534 | 0.778 |
|  |  | Cord blood IL-6 (pg/ml) | -0.034 | -0.083 | 0.016 | 0.175 |
|  |  | Cord blood TNF-α (pg/ml) | -0.537 | -2.099 | 1.025 | 0.486 |
| BMI (kg/m2) | Maternal Predictors | CRP at the 1st GDM visit (mg/L) | -0.059 | -0.134 | 0.016 | 0.125 |
|  |  | IL-6 at the 1st GDM visit (pg/ml) | -0.157 | -0.337 | -0.023 | 0.087 |
|  |  | TNF-α at the 1st GDM visit (pg/ml) | -0.641 | -1.003 | -0.279 | 0.001 |
|  | Fetal Predictors | Cord blood CRP (mg/L) | -2.566 | -3.666 | -1.465 | 0.000 |
|  |  | Cord blood IL-6 (pg/ml) | 0.000 | -0.071 | 0.071 | 0.997 |
|  |  | Cord blood TNF-α (mg/ml) | -2.177 | -3.671 | -0.684 | 0.006 |

Abbreviations: *BMI* body mass index; *CI* Confidence Interval; *CRP* C-reactive protein; *GDM* gestational diabetes mellitus; *IL-6* interleukin 6; *TNF-a* tumor necrosis factor alpha.

Univariate linear and logistic regression analyses at 1 year, adjusted for group allocation (intervention/usual care) and infant sex and age (where appropriate).
